# 1Infrared Absorption Spectrum of Methane From 2470 to 3200 cm^−1^

**DOI:** 10.6028/jres.064A.021

**Published:** 1960-06-01

**Authors:** Earle K. Plyler, Eugene D. Tidwell, Lamdin R. Blaine

## Abstract

The absorption spectrum of methane has been measured from 2470 to 3200 cm^−1^ with a high-resolution infrared spectrometer. Pressures from 0.01 to 4.5 cm of the gas were used in order to observe both intense and weak lines. A total of 2,460 lines were measured in the entire region. The *v*_3_ band at 3018 cm^−1^ was measured at very slow scanning rates and the lines of the *P* and *R* branches were resolved into several components. The observed spectrum is shown in five figures.

## 1. Introduction

A number of researches have been made on the infrared spectrum of methane since the first interpretation of the observed spectrum was made by Dennison in 1925 [1].^2^ It is now well established that the tetrahedral model is in accord with all the measurements of the infrared and the Raman spectrum. There are four fundamental vibrations which occur at about 2914, 1534, 3018, and 1306 cm^−1^ which are designated as *v*_1_ *v*_2_, *v*_3_, and *v*_4_, respectively. For a rigid molecule of this symmetry only the *v*_3_ and *v*_4_ vibrations are active in the infrared, but on account of interactions *v*_2_ becomes active in the infrared spectrum. This band has been measured by Burgess, Bell, and Nielsen [2]. More recently it has been shown by Allen and Plyler [3] that the *v*_3_ vibration at 3018 cm^−1^ which is triply degenerate has a weak component with a *Q*(*F^+^*) branch at 3022 cm^−1^. The *Q*(*F*^−^) branch is also probably active, but it has not been observed and may be strongly overlapped by the strong *Q*(*F*°) branch.

When the *v*_3_ band of methane is measured with low resolution the lines of the *P* and *R* branches are observed as single. With better resolution lines having large *J’*s begin to split up into several components. This splitting was found by Nielsen [4] to be rather large in *v*_4_, and Childs and Jahn [5] explained the splitting as produced by the Coriolis interaction between *v*_2_ and *v*_4_. The rotational structure of *v*_3_ also shows splittings in the *P* and *R* branches, but the separation between the components is much less than in *v*_4_. With a resolution of 0.05 cm^−1^ the lines of the *P* branch with *J*≥ 2 and the lines of the *R* branch with *J*≥ 3 have all been split into components (see ref. 3), the lines from *P* 9 to 15 each showing four components. The present investigation was undertaken to ascertain if, with higher resolution, the rotational lines of the *P* and *R* branches of *v*_3_ could be split into a greater number of components and to search for other bands which may appear in the region 2470 to 3200 cm^−1^.

## 2. Experimental Method

There was available in the Radiometry Section of the Bureau an infrared spectrometer which has a resolution between 0.02 and 0.03 cm^−1^. The instrument is equipped with a 10,000 line/inch grating which has a ruled surface of 4¾ × 8 in. The grating is double-passed and the energy is detected by a cooled PbS cell. A full description of the instrument is given in a previous paper [6]. The spectra were measured by using the fringes of a Fabry-Perot interferometer to interpolate between standard lines. As this method of measurement has been previously described [7] the details will not be repeated.

**Figure 1:**
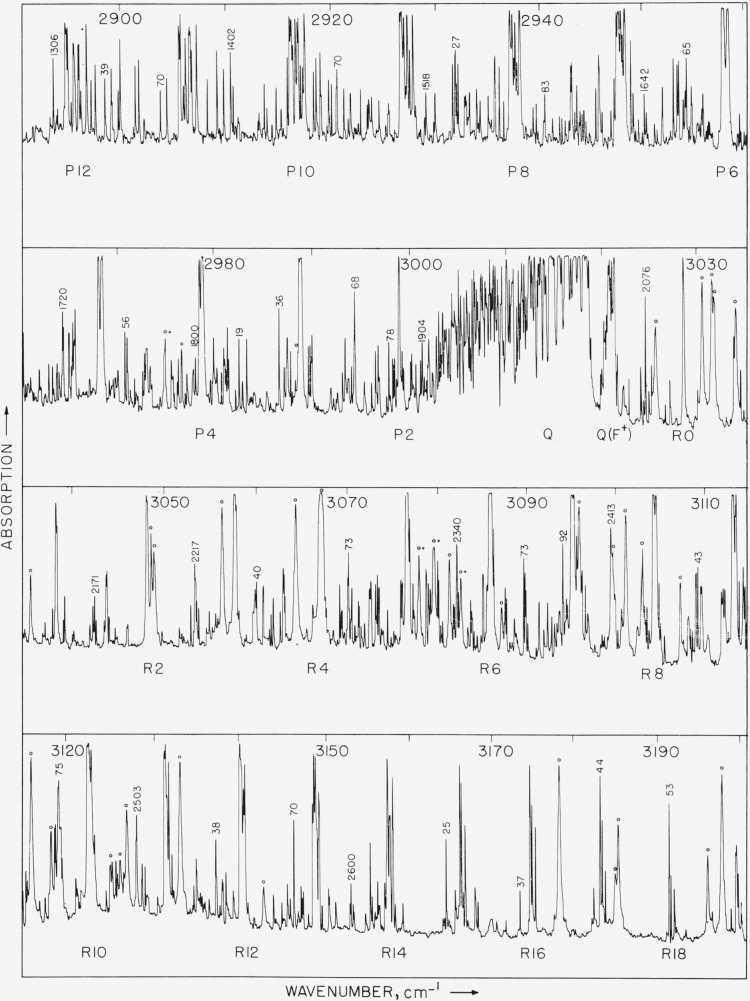
The infrared absorption of methane from 2890 to 3200 cm^−1^ as observed with a 10,000 lines/in grating used double pass. The gas pressure was 2 cm in a 6-m cell. The numbers on the lines correspond with those given in table 1.

**Figure 2:**
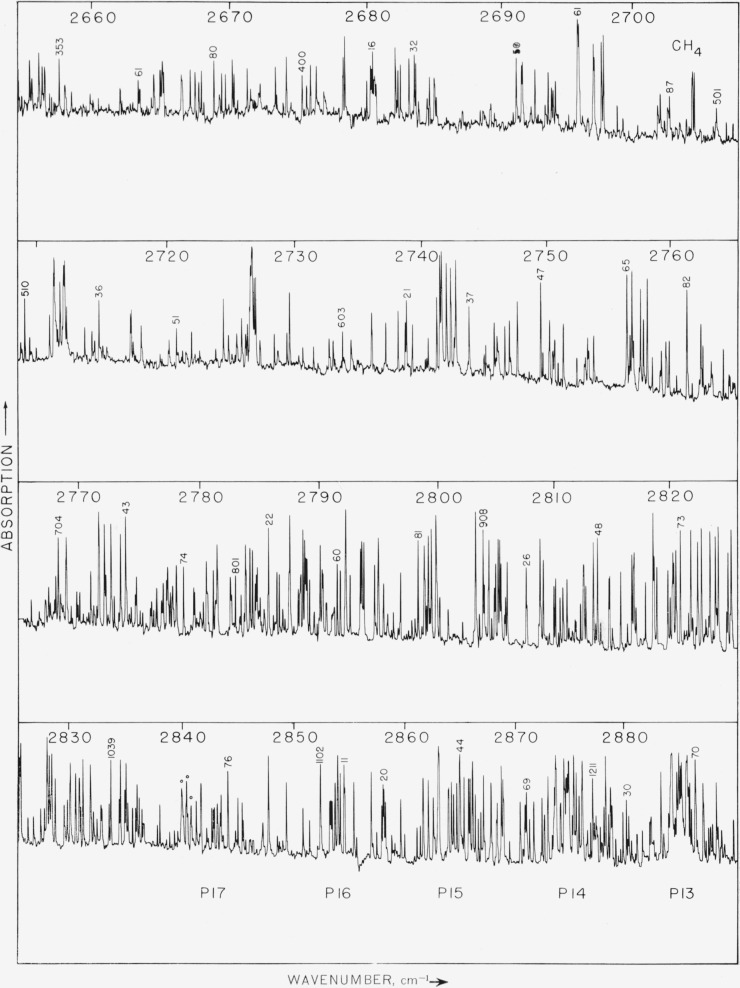
The observed spectrum of methane from 2655 to 2890 cm^−1^. The conditions of measurement were the same as for figure 1.

**Figure 3:**
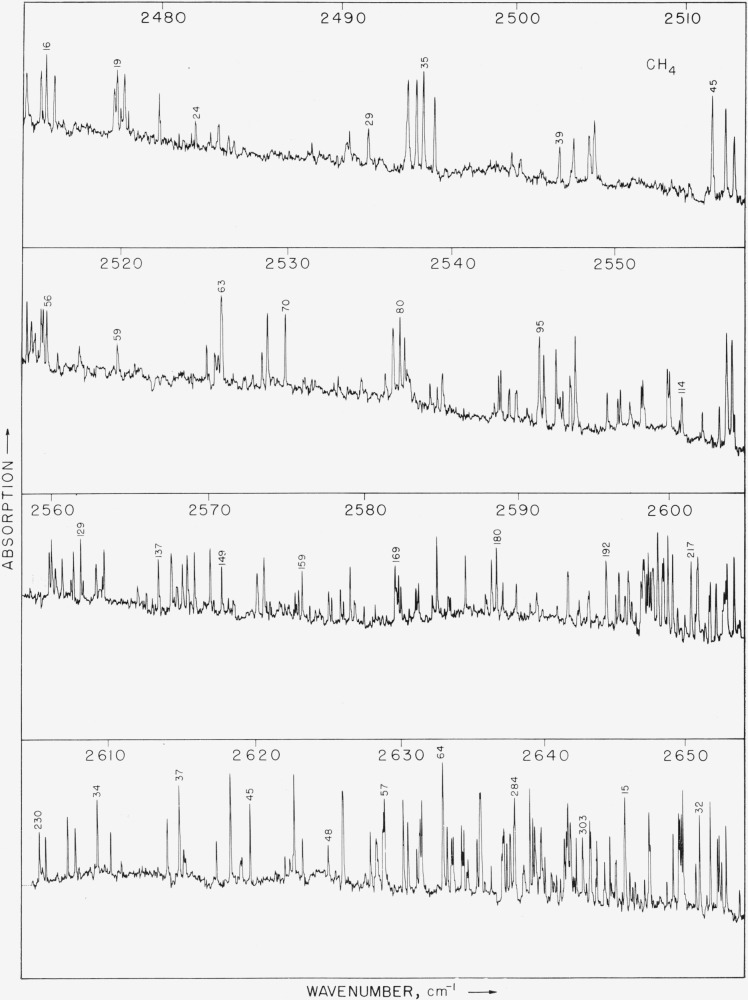
The observed spectrum of methane from 2470 to 2655 cm^−1^. The pressure of the gas was 4.5 cm. The other conditions of measurement were the same as for the spectra in figures 1 and 2.

The methane gas was obtained from the Matheson Company and the purity was given as 99 percent. No further purification of the gas was made. In the measurement of the spectra the gas pressure for different spectral regions was varied from 0.1 mm to 4.5 cm. The absorption cell had a path of 6 m which made it possible to use very low pressures in the regions of strong absorption. The spectrum was observed at various scanning speeds. In the regions where it was desired to bring out as much structure as possible, the spectrum was scanned at the rate of one wavenumber in 10 min, but in scanning the entire region from 2470 to 3200 cm^−1^ one wavenumber was recorded per minute. Even at this faster rate 12 hr were necessary and 32 ft of recorder paper were required for scanning the entire region. For the measuring charts a slower speed was used and 60 hr were required to cover the region included in figures 1, 2, and 3.

## 3. Experimental Results

Photographs of the recorder traces of the observed spectrum are shown in figures 1, 2, and 3. Figure 1 covers the region from 2890 to 3200 cm^−1^ and includes the intense component of from *R* 18 to *P* 12. The pressure of the gas in the cell was 2 cm for the spectrum in figures 1 and 2 and 4.5 cm for the results shown in figure 3. These pressures were selected in order to make it possible to observe the lines between the *P* and *R* branches of the main band.

About 2,650 lines were measured in the region from 2470 to 3200 cm^−1^ and serial numbers were placed on the figures which correspond with the line numbers given in table 1. This makes it possible to ascertain the wavenumber of any line in the observed spectrum.

**Figure 4:**
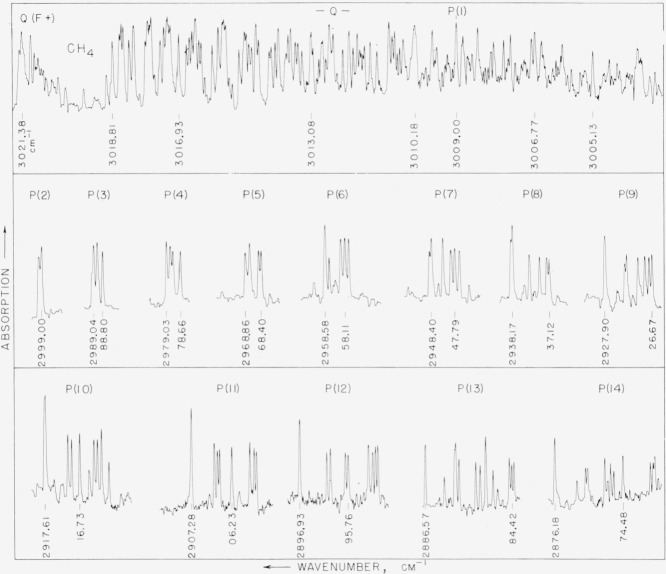
The *Q*(*F*^+^) and *Q*(*F*°) branches and the *P*(*F*^+^) branch of v_3_ of methane as measured with slow scanning speeds and a pressure 0.1 mm in a 6-m cell. The regions between consecutive *J*’s were omitted.

The amount of gas was too large and the scanning speed was too rapid to properly measure the components of the lines in the *P*, *Q*, and *R* branches of the most intense component of *v_3_*. In figure 4 is shown the spectrum of the *Q* branch and the *P* branch of this component as observed with 0.1 mm of pressure of methane.

**Figure 5:**
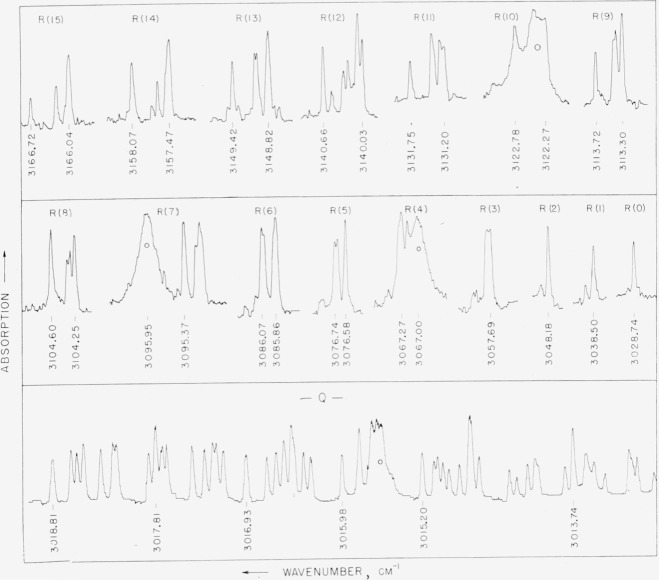
The *R*(*F*^−^) branch of methane as measured with a pressure 0.2 mm and the *Q*(*F*°) branch as measured with a pressure <0.1 mm.

The *Q*(*F*°) branch has very intense lines and the spectrum can be better represented by using less gas and scanning at a slower speed. The *Q* branch of this band, shown at the bottom strip of figure 5, was observed with a pressure between 0.1 and 0.01 mm and the scanning speed is one-third of that used in figure 4.

The two upper strips of figure 5 represent the *R*(*F*^−^) branch of the *v*_3_ band as observed with 0.2-mm pressure. The regions between the successive *R* lines were not recorded as at this low pressure very few lines were observed. Some lines of the atmospheric water band, 2*v*_2_, overlap this region and they are marked by small circles. The atmospheric water vapor lines are much broader than the methane lines on account of pressure broadening and they can be identified easily.

In table 2 the wavenumbers of the lines of the P, *Q*, and *R* branches are listed. This table applies to the experimental results shown in figures 4 and 5. The lines in the *P* and *R* branches are grouped under the *J* values so they can be related to the observed spectrum.

## 4. Discussion of Results

The spectrum of methane recorded in figures 1, 2, and 3 shows many lines which may be associated with several bands. The most prominent band is the allowed component of *v*_3_ which has *P*(*F*^+^),*Q*(*F*°), and *R*(*F*^−^) branches. The P, *Q*, and *R* branches of this band have been split into several components for each *J* as is shown by the experimental results in figures 4 and 5. Other normally forbidden transitions with components comprising the P(P°), P(*F*^−^), *Q*(*F*^−^), P(*F*°), and *R*(*F*^+^) branches occur in the same region as the strong component of *v*_3_, but with much less intensity. The *Q*(*F*^+^) branch is the prominent feature near 3021.18 cm^−1^. Other lines in this region may arise from isotopic molecules and from hot bands. Boyd and Thompson [8] showed the presence of a hot band in this region by measuring the absorption spectrum at elevated temperatures. There appears to be some experimental evidence for a weak *Q* branch at 3070 cm^−1^. The Raman spectra shows an intense line in this region and it has been classified as *2v_2_*. If the forbidden components of this band become active by interactions, there would be a total of two *Q* branches. A double type *Q* branch is observed in the region from 2820 to 2835 cm^−1^ which is one component of *v*_2_+*v*_4_. If all transitions are active there is a possibility for six components of this band. The *v*_1_ band, which arises from the totally symmetric vibration at 2914.4 cm^−1^, is the most intense in the Raman spectrum, but there is no evidence of a *Q* branch in this region of the observed spectrum. That is, at a pressure of 2 cm for a cell length of 6 m this band is not observed.

In figure 3 there is a concentration of lines near 2600 cm^−1^ which is a *Q* branch of 2*v*_4_. Since *v*_4_ is triply degenerate 2*v*_4_ could, with Coriolis interactions, consist of six separate subbands and may account for the large number of lines observed in the region from 2470 to 2650 cm^−1^.

Many features of the spectrum have been explained on the basis of the theoretical work of Jahn [9]. His calculations of the Coriolis interactions carried to a second order approximation have explained the splitting of the lines of the *P*, *Q*, and *R* branches into components. However his results are not adequate to give a quantitative agreement with the experimental results given in this paper. In order to properly account for the observed spectrum still higher order corrections must be included. A theoretical treatment of the spectrum of CH_4_, which has recently been carried out by Professor Karl Hecht of the University of Michigan, should make possible a better detailed analysis than is known at present. He has obtained excellent agreement with the experimental results for the *v*_3_ band for values of *J*⋜8, and a good agreement for values of *J*>8. His results will appear in a separate publication.

## Figures and Tables

**Table 1:** Vibration-rotation wavenumbers of methane from 2,450 to 3,200 cm^−1^

Line No.	cm^−1^	Line No.	cm^−1^	Line No.	cm^−1^	Line No.	cm^−1^	Line No.	cm^−1^	Line No.	cm^−1^
											
1	2450.87	60	2525.17	120	2557.55	181	2589.01	242	2618.90	300	2642.13
2	2451.10	61	2525.68	121	2559.83	182	2589.87	243	2618.94	301	2642.24
3	2452.32	62	2525.85	122	2560.00	184	2591.21	244	2619.03	302	2642.55
4	2452.87	63	2526.05	123	2560.20	185	2591.56	245	2619.59	303	2642.70
5	2459.59	64	2526.10	124	2560.57	186	2593.26	245a	2622.50	304	2642.98
6	2459.80	65	2527.38	125	2560.64	187	2593.91	246	2622.61	305	2643.22
7	2463.38	66	2527.46	126	2561.11	188	2593.99	247	2623.19	306	2643.29
8	2464.70	67	2527.94	127	2561.19	189	2594.60	248	2624.93	307	2643.64
9	2464.93	68	2528.46	128	2561.35	190	2594.65	249	2625.95	308	2643.72
10	2465.74	69	2528.81	129	2561.82	191	2595.18	250	2625.98	309	2643.82
11	2466.58	70	2529.88	130	2561.96	192	2595.79	251	2627.86	310	2644.18
12	2467.16	71	2530.96	131	2562.78	193	2596.43	252	2627.96	311	2644.24
13	2471.03	72	2531.08	132	2562.98	194	2596.66	253	2628.26	312	2644.65
14	2472.50	73	2531.46	133	2563.19	195	2597.09	254	2628.32	313	2645.04
15	2473.31	74	2533.09	134	2563.31	196	2597.28	255	2628.70	314	2645.09
16	2473.61	75	2534.53	135	2565.44	197	2597.34	256	2628.76	315	2645.73
17	2474.05	76	2535.99	136	2566.04	198	2597.52	257	2628.86	316	2646.04
18	2477.35	77	2536.45	137	2566.82	199	2598.13	258	2630.17	317	2646.24
19	2477.51	78	2536.49	138	2567.65	200	2598.27	259	2630.48	318	2646.40
20	2477.72	79	2536.68	139	2567.81	201	2598.36	260	2631.08	319	2646.58
21	2477.91	80	2536.88	140	2567.95	202	2598.53	261	2631.27	320	2646.84
22	2478.12	81	2537.01	141	2568.02	203	2598.65	262	2631.34	321	2647.08
23	2479.86	82	2537.17	142	2568.30	204	2598.76	263	2631.47	322	2647.31
24	2481.86	83	2537.33	143	2568.61	205	2598.88	264	2632.93	323	2647.42
25	2483.16	84	2537.39	144	2568.72	206	2598.92	265	2633.21	324	2647.50
26	2483.70	85	2538.72	145	2569.10	207	2598.97	266	2633.44	325	2648.66
27	2490.32	86	2539.17	146	2569.64	208	2599.27	267	2633.52	326	2649.14
28	2490.50	87	2539.48	147	2570.10	209	2599.52	268	2633.62	327	2649.52
29	2491.62	88	2542.94	148	2570.31	210	2599.57	269	2634.21	328	2649.61
30	2493.09	89	2543.08	149	2570.83	211	2599.64	270	2634.30	329	2649.71
31	2493.70	90	2543.55	150	2573.06	212	2599.79	271	2634.39	330	2649.83
32	2493.82	91	2543.92	151	2573.46	213	2599.93	272	2634.58	331	2650.74
33	2493.90	92	2544.00	152	2573.53	214	2600.27	273	2634.66	332	2651.02
34	2494.38	93	2545.28	153	2573.73	215	2600.58	274	2635.29	333	2651.50
35	2494.77	94	2545.35	154	2573.92	216	2601.06	275	2635.50	334	2651.81
36	2495.40	95	2545.39	155	2575.12	217	2601.49	276	2635.56	335	2652.29
37	2499.81	96	2545.68	156	2575.50	218	2601.86	277	2636.31	336	2652.42
38	2500.30	97	2546.42	157	2575.60	219	2601.92	278	2636.99	337	2652.59
39	2502.58	98	2546.54	158	2575.73	221	2602.66	279	2637.07	338	2652.92
40	2503.40	99	2546.66	159	2575.98	222	2602.76	280	2637.14	339	2653.85
41	2504.30	100	2546.84	160	2576.46	223	2603.02	281	2637.33	340	2654.58
42	2504.38	101	2547.12	161	2576.82	224	2603.13	282	2637.58	341	2654.72
43	2504.58	102	2547.27	162	2577.11	225	2603.57	283	2637.85	342	2654.98
44	2504.63	103	2547.36	163	2577.70	226	2603.66	284	2637.90	343	2655.54
45	2511.55	104	2547.62	164	2577.89	227	2603.77	285	2638.45	344	2655.68
46	2512.33	105	2549.59	166	2578.48	228	2603.86	286	2638.52	345	2655.85
47	2512.84	106	2550.26	167	2578.67	229	2604.32	287	2638.97	346	2655.96
47a	2512.92	107	2550.42	168	2579.09	230	2605.34	288	2639.18	347	2656.14
48	2514.50	108	2551.03	169	2582.01	230a	2605.52	289	2639.30	348	2656.21
49	2514.67	109	2551.74	170	2582.08	231	2605.75	290	2639.37	349	2656.36
50	2514.75	110	2551.84	171	2582.22	232	2607.25	290a	2639.60	350	2656.44
51	2514.90	111	2553.38	172	2582.39	233	2607.75	291	2639.71	351	2656.62
52	2514.96	112	2553.50	173	2583.35	234	2609.25	292	2639.76	352	2656.65
53	2515.14	113	2553.58	174	2583.52	235	2610.16	293	2639.83	353	2657.71
54	2515.31	114	2554.28	175	2584.42	236	2614.16	294	2640.00	354	2658.11
55	2515.45	115	2555.54	176	2584.73	237	2614.77	295	2641.41	355	2658.58
56	2515.66	116	2555.64	177	2686.59	238	2615.06	296	2641.54	356	2659.90
57	2516.28	117	2556.60	178	2588.30	239	2615.18	297	2641.66	357	2662.06
58	2517.58	118	2557.08	179	2588.40	240	2617.29	298	2641.85	358	2662.10
59	2519.84	119	2557.42	180	2588.64	241	2618.27	299	2642.03	359	2663.01
360	2663.28	450	2691.20	530	2712.94	619	2738.14	702	2767.96	784	2780.26
361	2663.38	451	2691.39	531	2713.05	620	2738.68	703	2768.06	785	2780.53
362	2663.48	452	2691.59	532	2713.66	621	2738.79	704	2768.27	786	2780.60
364	2664.51	353	2691.66	533	2714.22	622	2739.26	705	2768.45	787	2780.64
366	2664.77	453a	2692.31	534	2714.35	623	2740.24	705a	2768.52	788	2780.73
367	2664.96	454	2692.64	535	2714.43	624	2740.36	706	2768.59	789	2780.90
368	2665.07	455	2693.40	536	2714.76	625	2740.48	707	2768.64	790	2781.08
369	2665.16	456	2693.62	537	2715.06	626	2741.19	708	2768.91	791	2781.21
370	2665.27	457	2693.88	538	2716.84	627	2741.44	709	2768.94	792	2781.26
371	2666.28	458	2693.99	539	2717.07	628	2741.58	710	2769.05	793	2781.40
372	2666.47	459	2694.17	540	2717.21	629	2741.96	711	2769.21	794	2781.48
373	2666.53	460	2694.37	541	2717.29	630	2742.28	712	2769.32	795	2781.93
374	2667.13	461	2695.82	542	2717.42	631	2742.35	713	2769.43	796	2782.37
375	2667.46	462	2695.85	543	2717.95	632	2742.55	714	2769.52	797	2782.51
376	2667.74	463	2695.89	544	2718.05	633	2742.60	715	2769.67	798	2782.61
377	2667.94	464	2695.95	545	2718.13	634	2742.73	716	2769.73	799	2782.71
378	2668.10	465	2696.01	546	2718.20	636	2743.68	717	2769.78	800	2782.77
379	2668.24	466	2696.24	547	2719.46	637	2743.78	718	2769.88	801	2782.93
380	2668.85	467	2696.55	548	2719.62	638	2744.94	719	2770.04	802	2783.22
383	2669.25	468	2696.64	549	2720.10	639	2745.08	720	2770.20	803	2783.28
384	2669.41	469	2696.95	550	2720.15	640	2745.72	721	2770.32	804	2783.34
385	2669.70	470	2697.00	551	2720.78	641	2745.84	722	2770.45	805	2783.42
386	2669.92	471	2697.06	552	2720.90	642	2745.93	723	2770.62	806	2783.68
387	2670.20	472	2697.12	553	2721.13	643	2746.04	724	2770.76	807	2783.78
388	2670.32	473	2697.49	554	2721.23	644	2746.57	725	2770.82	808	2783.83
390	2670.54	474	2697.66	555	2721.38	645	2746.95	726	2770.94	809	2784.08
391	2671.28	475	2697.81	556	2721.49	646	2747.61	727	2771.05	810	2784.15
392	2671.53	376	2698.83	557	2721.78	647	2749.49	728	2771.18	811	2784.36
394	2672.12	477	2699.19	558	2721.90	648	2749.66	729	2771.27	812	2784.58
395	2672.22	478	2699.26	559	2721.94	649	2750.16	730	2771.43	813	2784.67
398	2673.35	478a	2700.37	560	2722.10	650	2750.20	731	2771.60	814	2784.76
399	2674.15	479	2701.51	561	2722.20	651	2750.45	732	2771.67	815	2784.90
400	2675.28	480	2701.61	562	2722.42	652	2750.62	733	2772.09	816	2785.01
401	2675.64	481	2701.90	563	2723.79	653	2750.88	734	2772.21	817	2785.10
402	2675.91	482	2702.03	564	2723.90	654	2751.31	735	2772.56	818	2785.20
403	2676.27	483	2702.10	565	2724.26	655	2752.40	736	2772.63	819	2785.26
404	2676.34	484	2702.40	566	2724.35	656	2752.60	737	2772.73	820	2785.32
405	2676.92	385	2702.66	567	2724.40	657	2752.99	738	2772.92	821	2785.68
406	2677.01	486	2702.72	568	2724.79	658	2753.07	739	2773.26	822	2785.72
407	2677.64	487	2702.81	569	2725.04	659	2753.24	740	2773.43	823	2786.10
408	2678.30	488	2703.31	570	2725.29	660	2753.33	741	2773.71	824	2786.26
409	2678.34	489	2703.59	571	2725.40	663	2753.75	742	2773.77	825	2786.42
410	2678.48	490	2704.11	572	2725.48	664	2756.37	743	2773.84	826	2786.63
411	2678.70	491	2704.32	573	2725.81	665	2756.46	744	2773.96	827	2786.82
412	2679.20	492	2704.56	574	2726.12	666	2756.61	745	2774.14	828	2786.88
413	2680.00	493	2704.61	575	2726.27	667	2756.71	746	2774.32	829	2787.07
414	2680.37	494	2704.73	576	2726.43	668	2756.88	747	2774.65	830	2787.21
415	2680.46	495	2704.82	577	2726.50	669	2756.98	748	2774.73	831	2787.28
416	2680.55	496	2705.13	578	2726.63	670	2757.14	749	2774.94	832	2787.44
417	2680.64	499	2706.17	580	2726.68	671	2757.46	750	2775.05	833	2787.51
418	2680.68	500	2706.34	581	2726.79	672	2757.58	751	2775.30	834	2787.84
419	2680.80	501	2706.40	582	2726.91	673	2757.82	752	2775.52	835	2788.19
420	2681.37	502	2706.50	583	2727.25	674	2758.13	753	2775.93	836	2788.32
421	2681.56	503	2706.76	584	2728.37	675	2758.54	754	2776.02	837	2788.39
422	2682.23	504	2707.15	585	2728.63	676	2759.10	755	2776.18	838	2788.43
423	2682.35	505	2707.28	586	2728.69	677	2759.18	756	2776.38	839	2788.60
424	2682.42	506	2707.64	587	2729.34	678	2759.27	757	2776.44	840	2788.72
425	2682.61	507	2708.52	588	2729.56	679	2759.63	758	2776.78	841	2788.76
426	2682.72	508	2708.73	589	2730.10	680	2759.70	759	2776.92	842	2788.84
427	2682.86	509	2708.82	590	2730.29	681	2759.92	760	2777.10	843	2788.94
429	2683.25	510	2709.05	591	2730.61	682	2761.35	761	2777.18	844	2789.15
430	2683.45	511	2709.41	592	2730.70	683	2762.34	762	2777.28	845	2789.55
431	2683.52	512	2709.51	593	2713.46	684	2762.47	763	2777.31	846	2789.66
432	2683.64	513	2709.91	594	2731.80	685	2762.65	764	2777.39	847	2789.77
433	2683.75	514	2710.08	597	2732.68	686	2763.33	765	2777.51	848	2789.93
434	2683.95	515	2710.96	598	2733.06	687	2763.40	766	2777.55	849	2790.06
435	2684.58	516	2711.27	599	2733.13	688	2764.30	767	2777.69	850	2790.23
436	2684.64	517	2711.32	601	2733.56	689	2764.74	768	2777.74	851	2790.30
437	2684.74	518	2711.41	602	2733.68	690	2764.83	769	2777.84	852	2790.42
438	2685.12	519	2711.46	603	2733.74	691	2765.06	770	2777.95	853	2790.56
439	2685.25	520	2711.49	604	2733.83	692	2765.20	771	2778.04	854	2790.60
441	2687.18	521	2711.58	605	2733.88	693	2765.72	774	2778.64	856	2791.03
442	2688.52	522	2711.71	606	2733.94	694	2766.02	776	2779.48	857	2791.10
443	2688.72	523	2711.76	607	2734.44	695	2766.68	777	2779.57	858	2791.17
444	2689.17	524	2711.95	610	2734.90	696	2767.18	778	2779.72	859	2791.24
445	2689.25	525	2712.05	611	2735.00	697	2767.23	779	2779.76	860	2791.47
446	2689.33	526	2712.11	613	2735.44	698	2767.48	780	2779.87	861	2791.75
447	2689.53	527	2712.14	614	2736.04	699	2767.62	781	2779.98	862	2792.14
448	2689.60	528	2712.27	617	2737.04	700	2767.70	782	2780.07	863	2792.22
449	2690.96	529	2712.36	618	2737.16	701	2767.86	783	2780.20	864	2792.24
865	2792.58	948	2813.72	1049	2836.48	1208	2876.66	1351	2901.05	1497	2925.62
865a	2793.38	949	2813.99	1049b	2836.67	1210	2876.95	1352	2901.30	1511	2928.14
866	2793.44	951	2814.70	1049d	2837.75	1211	2877.15	1353	2901.50	1519	2928.22
867	2793.55	952	2814.79	1049e	2837.97	1212	2877.29	1354	2901.85	1513	2928.42
868	2793.58	953	2815.00	1049h	2839.29	1213	2877.36	1355	2902.23	1514	2928.50
869	2793.68	954	2815.74	1051	2839.91	1214	2877.47	1356	2902.28	1515	2928.59
870	2793.74	955	2816.32	1052	2839.96	1215	2877.53	1357	2902.34	1516	2928.98
871	2794.64	957	2816.70	1053	2840.00	1216	2877.62	1358	2902.53	1517	2929.01
872	2794.76	958	2816.84	1054	2840.10	1217	2877.84	1368	2903.60	1518	2929.14
872a	2794.94	959	2816.90	1055	2840.33	1218	2877.97	1370	2903.83	1520	2929.53
873	2794.97	960	2817.06	1056	2840.36	1219	2878.02	1373	2904.48	1599	2929.74
875	2795.41	962	2817.88	1057	2840.46	1220	2878.16	1374	2905.07	1523	2929.89
876	2795.69	963	2818.55	1059	2840.71	1221	2878.33	1391	2907.56	1524	2930.01
877	2795.75	964	2818.63	1060	2840.75	1222	2878.58	1392	2907.92	1595	2931.41
878	2795.93	965	2818.86	1063	2841.21	1223	2878.62	1393	2908.29	1526	2931.72
879	2796.06	966	2819.82	1064	2841.61	1224	2878.81	1394	2908.52	1527	2931.96
880	2796.20	967	2820.03	1066	2842.14	1225	2878.84	1395	2908.72	1528	2932.08
880b	2796.62	968	2820.20	1068	2842.59	1226	2878.93	1396	2908.88	1529	2932.22
880c	2796.83	969	2820.26	1069	2842.72	1227	2879.26	1397	2909.23	1530	2932.87
880e	2797.10	970	2820.33	1070	2842.80	1228	2879.38	1399	2909.54	1531	2932.96
880f	2797.32	971	2820.51	1071	2843.06	1229	2879.93	1401	2909.85	1532	2932.99
880g	2797.52	973	2820.90	1072	2843.30	1230	2880.27	1402	2910.52	1533	2933.04
880h	2797.77	977	2821.59	1073	2843.33	1231	2880.39	1403	2910.62	1534	2933.13
880i	2797.84	978	2821.81	1074	2843.41	1232	2880.49	1404	2910.78	1535	2933.18
880j	2798.06	979	2821.94	1076	2844.01	1233	2880.70	1405	2910.95	1537	2933.33
881	2798.31	983	2822.37	1077	2844.49	1234	2880.86	1406	2911.15	1539	2933.59
882	2798.49	984	2822.47	1078	2844.56	1235	2881.07	1407	2911.24	1540	2933.70
883	2798.62	985	2822.72	1079	2844.67	1236	2881.22	1410	2911.59	1541	2934.01
884	2798.81	987	2823.05	1081	2844.91	1237	2881.26	1420	2912.70	1542	2934.08
885	2798.87	990	2823.34	1082	2845.30	1238	2881.73	1423	2913.08	1543	2934.17
886	2799.03	991	2823.46	1083	2845.40	1241	2882.39	1424	2913.15	1544	2934.20
887	2799.18	993	2823.89	1093	2847.14	1242	2882.47	1425	2913.27	1545	2931.72
888	2799.32	994	2824.00	1094	2847.68	1243	2882.52	1426	2913.32	1546	2931.96
889	2799.42	995	2824.14	1095	2848.48	1244	2882.69	1427	2913.58	1548	2934.33
892	2799.81	996	2824.86	1096	2849.29	1245	2882.78	1428	2913.76	1549	2934.38
893	2799.87	997	2824.99	1097	2850.79	1247	2882.97	1429	2913.81	1550	2934.94
894	2799.94	998	2825.19	1098	2851.38	1249	2883.42	1431	2914.02	1551	2935.13
896	2800.15	999	2825.28	1114	2855.61	1250	2883.68	1432	2914.47	1552	2935.25
898	2800.34	1001	2825.48	1115	2856.94	1251	2884.13	1433	2914.89	1554	2935.37
899	2800.84	1002	2825.60	1116	2857.10	1252	2884.28	1434	2915.24	1555	2935.44
900	2801.30	1003	2825.66	1117	2857.44	1271	2886.74	1435	2915.30	1556	2935.76
902	2802.10	1004	2825.74	1119	2857.90	1272	2887.30	1436	2915.39	1557	2935.81
904	2803.10	1005	2825.78	1120	2858.00	1273	2887.37	1437	2915.56	1572	2936.20
905	2803.21	1006	2826.35	1121	2858.11	1274	2887.57	1452	2917.68	1573	2936.63
907	2803.55	1007	2826.86	1122	2858.24	1275	2887.80	1453	2917.83	1574	2938.77
908	2803.84	1008	2827.49	1124	2858.78	1276	2887.95	1455	2918.03	1576	2935.44
909	2803.94	1009	2827.83	1126	2859.60	1277	2888.01	1456	2918.46	1578	2938.82
911	2804.19	1010	2828.06	1127	2859.96	1278	2888.18	1457	2918.1	1579	2938.93
912	2804.25	1010a	2828.11	1128	2861.09	1279	2888.31	1459	2919.09	1580	2939.27
913	2804.30	1011	2828.25	1129	2861.37	1280	2888.52	1460	2919.19	1581	2939.49
914	2804.46	1012	2828.40	1130	2861.61	1281	2888.66	1463	2919.70	1582	2939.61
915	2804.55	1013	2828.48	1131	2861.65	1281a	2888.70	1464	2919.88	1583	2939.80
917	2804.76	1014	2828.77	1148	2865.90	1283	2888.92	1465	2919.97	1584	2939.98
918	2804.86	1015	2829.56	1149	2866.11	1284	2889.03	1466	2920.16	1585	2940.50
919	2805.07	1018	2829.86	1151	2866.35	1291	2890.12	1467	2920.27	1587	2940.64
920	2805.23	1020	2830.07	1152	2866.65	1293	2890.63	1469	2920.58	1588	2940.98
921	2805.35	1021	2830.13	1153	2866.84	1296	2891.32	1470	2920.65	1589	2942.08
923	2805.66	1022	2830.58	1155	2867.09	1300	2891.90	1471	2921.30	1590	2942.33
924	2805.75	1023	2830.89	1156	2867.74	1304	2892.57	1472	2921.70	1591	2942.65
925	2805.90	1024	2830.94	1158	2868.21	1305	2893.51	1473	2921.91	1593	2942.76
926	2807.54	1025	2831.05	1159	2868.27	1306	2893.82	1474	2921.95	1594	2943.03
927	2807.61	1026	2831.25	1160	2868.69	1308	2894.07	1475	2922.10	1595	2943.12
928	2808.62	1027	2831.90	1161	2868.82	1309	2894.14	1476	2922.29	1596	2942.08
929	2808.73	1028	2831.95	1162	2868.90	1310	2894.22	1477	2922.44	1597	2943.19
930	2808.79	1029	2832.00	1164	2869.44	1312	2894.36	1478	2922.63	1599	2943.30
931	2809.62	1030	2832.16	1165	2870.35	1313	2894.42	1479	2922.88	1600	2943.59
933	2809.93	1032	2832.49	1166	2870.40	1314	2894.53	1480	2923.37	1601	2943.66
934	2810.67	1034	2832.86	1168	2870.87	1335	2897.35	1481	2923.42	1602	2943.78
935	2810.34	1035	2832.96	1169	2870.99	1336	2897.57	1482	2923 53	1693	2943.78
936	2810.48	1037	2833.56	1171	2871.30	1338	2897.75	1483	2923.65	1604	2943.99
937	2810.71	1039	2833.73	1173	2871.68	1339	2898.65	1484	2923.71	1605	2944.12
938	2811.05	1040	2834.45	1174	2872.32	1340	2899.23	1485	2923.77	1606	2944.29
939	2811.20	1041	2834.57	1175	2872.44	1341	2899.28	1486	2923.83	1607	2944.46
940	2811.75	1042	2834.94	1176	2872.67	1342	2899.36	1487	2923.91	1608	2944.50
942	2811.24	1043	2835.04	1178	2872.96	1343	2899.48	1488	2924.00	1609	2944.75
943	2812.48	1044	2835.23	1179	2873.01	1345	2899.88	1490	2924.62	1610	2945.01
944	2812.54	1045	2835.63	1180	2873.39	1346	2900.08	1491	2924.77	1611	2945.20
945	2812.69	1046	2835.90	1181	2873.53	1347	2900.28	1493	2925.27	1612	2945.40
946	2813.35	1047	2836.01	1206	2876.53	1348	2900.43	1495	2925.49	1613	2945.51
947	2813.55	1048	2836.22	1207	2876.62	1350	2900.67	1496	2925 56	1614	2945.62
1615	2945.92	1703	2962.51	1790	2976.56	1887	2999.12	2139	3034.24	2250	3063.20
1616	2945.95	1704	2962.64	1791	2976.66	1888	2999.20	2140	3034.32	2251	3064.19
1617	2946.05	1705	2962.70	1792	2976.79	1889	2999.32	2141	3034.48	2252	3064.40
1518	2946.25	1706	2962.87	1794	2977.16	1890	2999.46	2142	3034.68	2253	3064.50
1619	2946.48	1707	2963.00	1795	2977.34	1891	2999.68	2143	3034.78	2254	3065.58
1620	2946.65	1708	2963.10	1797	2977.87	1892	2999.81	2144	3034.81	2255	3066.28
1621	2947.09	1709	2963.15	1803	2978.21	1893	3000.00	2145	3035.14	2256a	3067.00
1633	2948.56	1710	2963.42	1801	2979.38	1895	3000.17	2146	3035.61	2257	3067.64
1634	3948.73	1711	2963.48	1802	2979.51	1896	3000.31	2147	3035.77	2258	3067.76
1635	2949.10	1712	2963.63	1803	2979.74	1897	3000.44	2152	3037.26	2259	3068.04
1636	2949.24	1713	2963.86	1804	2979.77	2021	3019.00	2153	3037.33	2260	3068.15
1637	2949.32	1714	2964.95	1805	2979.95	2022	3019.10	2155	3037.57	2261	3068.31
1638	2949.53	1715	2064.26	1806	2980.03	2023	3019.18	2157	3037.93	2262	3068.52
1639	2949.81	1716	2964.31	1807	2980.07	2024	3019.31	2158	3038.13	2262a	3068.59
1640	2949.89	1717	2965.51	1808	2980.19	2025	3019.47	2162	3038.69	2263	3068.98
1641	2950.13	1718	2964.60	1809	2980.39	2026	3019.58	2163	3039.27	2264	3069.16
1642	2950.33	1719	2964.78	1810	2980.59	2027	3019.62	2166	3039.42	2265	3069.24
1643	2950.52	1720	2964.86	1811	2980.73	2028	3019.67	2167	3042.06	2266	3069.42
1644	2950.65	1721	2964.94	1812	2980.84	2029	3019.81	2170	3042.41	2267	3069.54
1645	2950.85	1722	2965.09	1813	2981.08	2030	3019.88	2171	3042.57	2268	3069.63
1646	2951.30	1723	2965.28	1814	2981.17	2031	3020.02	2172	3042.73	2269	3069.72
1647	2951.38	1724	2965.45	1815	2981.31	2032	3020.15	2173	3043.59	2270	3069.79
1648	2951.56	1725	2965.51	1816	2981.46	2033	3020.22	2174	3043.74	2271	3070.01
1649	2952.04	1726	2965.59	1817	2981.59	2034	3020.38	2175	3043.79	2272	3070.13
1650	2952.11	1727	2965.74	1818	2982.26	2035	3020.46	2176	3043.90	2273	3070.22
1651	2952.40	1727a	2965.83	1819	2982.64	2036	3020.55	2177	3043.97	2274	3070.32
1652	2952.46	1728	2965.86	1820	2982.98	2037	3020.67	2178	3044.11	2275	3070.48
1653	2952.65	1729	2965.96	1821	2983.38	2038	3020.78	2179	3044.17	2276	3070.58
1654	2952.77	1730	2966.00	1822	2983.45	2039	3020.85	2180	3044.56	2277	3070.61
1655	2952.87	1731	2966.10	1823	2983.61	2040	3020.93	2181	3044.78	2278	3070.93
1656	2953.08	1732	2966.46	1824	2983.69	2041	3021.06	2182	3044.95	2279	3071.10
1657	2953.18	1733	2966.56	1825	2983.81	2042	3021.17	2183	3045.98	2281	3071.31
1658	2953.40	1734	2966.62	1827	2984.08	2043	3021.30	2184	3046.04	2282	3071.41
1659	2953.53	1735	2966.78	1828	2984.16	2044	3021.38	2185	3046.10	2283	3071.62
1660	2953.67	1736	2966.86	1831	2985.36	2045	3021.46	2186	3047.91	2284	3071.72
1661	2953.71	1737	2967.03	1832	2985.42	2046	3021.49	2190	3048.58	2285	3072.00
1663	2954.15	1738	2967.10	1836	2986.70	2047	3021.63	2191	3048.65	2286	3072.06
1664	2954.34	1739	2967.23	1837	2987.22	2048	3021.78	2192	3048.77	2287	3072.53
1665	2954.44	1740	2967.50	1838	2987.43	2049	3021.82	2193	3048.94	2288	3072.57
1666	2954.54	1741	2967.70	1839	2987.53	2051	3022.31	2194	3049.01	2289	3072.66
1667	2954.60	1742	2967.85	1840	2987.70	2052	3022.37	2196	3049.80	2290	3072.71
1668	2954.70	1744	2969.10	1841	2987.88	2053	3022.43	2198	3050.17	2291	3073.10
1669	2954.76	1745	2969.28	1842	2988.03	2056	3022.69	2199	3050.29	2292	3073.21
1670	2954.91	1746	2969.46	1843	2988.13	2057	3022.82	2204	3051.71	2293	3073.30
1671	2955.00	1747	2969.64	1844	2988.18	2058	3022.93	2206	3051.92	2294	3073.42
1672	2955.17	1748	2969.70	1845	2988.34	2059	3023.03	2209	3052.19	2295	3073.58
1673	2955.42	1749	2969.78	1846	2989.69	2060	3023.12	2211	3052.64	2296	3073.63
1674	2955.47	1750	2969.89	1847	2989.88	2073	3024.28	2213	3052.93	2298	3073.85
1675	2955.54	1751	2970.07	1848	2989.98	2074	3024.42	2216	3053.28	2299	3073.99
1676	2955.69	1752	2970.12	1849	2990.08	2075	3024.56	2217	3053.39	2300	3074.06
1677	2955.76	1753	2970.24	1850	2990.22	2076	3024.72	2218	3053.46	2301	3074.55
1678	2955.93	1754	2970.32	1851	2990.36	2077	3025.07	2219	3053.49	2302	3074.90
1679	2955.98	1755	2970.38	1852	2990.70	2080	3025.35	2220	3053.60	2303	3075.07
1680	2956.08	1756	2971.06	1853	2991.92	2081	3025.46	2221	3053.83	2304	3075.22
1680a	2956.13	1757	2971.27	1854	2991.99	2082	3025.57	2223	3054.64	2305	3075.41
1680b	2956.30	1758	2971.34	1855	2992.27	2083	3025.68	2225	3054.97	2306	3075.99
1680c	2956.39	1759	2971.59	1856	2992.53	2084	3025.73	2226	3055.06	2307	3076.13
1680d	2956.52	1761	2972.02	1857	2992.67	2085	3025.77	2227	3055.33	2308	3076.17
1680e	2956.63	1762	2972.15	1859	2993.19	2086	3025.82	2228	3055.65	2309	3076.36
1689f	2956.74	1763	2972.36	1860	2993.47	2087	3025.87	2229	3055.89	2313	3077.16
1681	2959.50	1767	2972.83	1861	2993.53	2088	3025.96	2231	3056.34	2314	3077.36
1682	2959.60	1768	2973.01	1862	2993.63	2089	3026.07	2231a	3056.53	2315	3077.44
1683	2959.73	1769	2973.18	1863	2993.73	2092	3026.35	2232	3057.90	2316	3077.53
1684	2959.85	1770	2973.23	1864	2993.84	2097	3026.79	2233	3058.18	2318	3077.87
1686	2960.20	1771	2973.56	1866	2994.14	2098	3026.94	2234	3058.80	2319	3077.95
1687	2960.38	1772	2973.62	1867	2994.29	2102	3027.30	2235	3058.95	2320	3078.02
1688	2960.66	1773	2973.77	1868	2994.46	2107	3027.88	2236	3059.47	2321	3078.12
1689	2960.77	1774	2974.57	1871	2995.46	2111	3028.82	2237	3059.73	2322	3078.77
1690	2960.86	1775	2974.83	1873	2996.16	2114	3029.17	2238	3059.89	2323	3078.82
1691	2960.94	1777	2975.01	1874	2996.60	2122	3030.21	2239	3059.94	2324	3079.04
1692	2961.14	1778	2975.09	1875	2996.88	2125	3030.68	2240	3060.10	2325	3079.13
1693	2961.32	1779	2975.13	1876	2996.99	2126	3030.73	2241	3060.79	2326	3079.27
1694	2961.43	1781	1975.68	1877	2997.71	2127	3030.85	2242	3060.84	2327	3079.44
1695	2961.65	1782	2975.74	1878	2997.96	2131	3031.71	2243	3061.42	2328	3079.58
1697	2961.85	1783	2975.78	1879	2998.10	2132	3031.77	2244	3061.49	2329	3079.66
1698	2961.99	1784	2975.86	1880	2998.27	2133	3031.91	2245	3061.69	2329a	3079.72
1699	2962.13	1785	2975.93	1881	2998.6	2134	3032.04	2246	3061.91	2330	3080.05
1700	2962.19	1786	2976.03	1882	2998.45	2135	3032.14	2247	3062.68	2331	3080.18
1701	2962.31	1788	2976.33	1883	2998.58	2137	3033.98	2248	3063.00	2332	3080.42
1702	2962.43	1789	2976.45	1884	2998.78	2138	3034.06	2249	3063.10	2333	3080.62
2334	3081.10	2380	3091.29	2425	3103.40	2473	3118.64	2533	3135.93	2605	3155.42
2336	3081.35	2381	3091.46	2426	3103.59	2474	3118.94	2534	3136.41	2606	3155.63
2338	3081.82	2382	3092.00	2427	3103.77	2475	3119.01	2535	3136.61	2607	3155.99
2339	3081.92	2383	3092.40	2428	3104.90	2476	3119.16	2536	3136.88	2608	3156.16
2340	3082.17	2384	3092.70	2429	3105.08	2477	3119.41	2538	3137.26	2609	3156.32
2341	3082.22	2385	3092.84	2430	3105.52	2478	3119.51	2539	3137.34	2610	3156.47
2342	3082.35	2386	3092.89	2432	3107.33	2479	3120.95	2540	3137.70	2611	3156.54
2343	3082.52	2387	3093.21	2433	3107.65	2480	3121.10	2541	3137.82	2612	3156.72
2344	3082.62	2388	3093.29	2434	3107.74	2481	3121.18	2542	3137.97	2613	3156.88
2345	3082.71	2389	3093.47	2435	3107.90	2482	3121.33	2543	3138.08	2614	3157.15
2346	3082.82	2390	3093.68	2436	3108.17	2484	3123.11	2544	3138.44	2615	3157.40
2347	3083.41	2391	3093.76	2437	3108.24	2485	3123.66	2545	3138.52	2620	3158.14
2348	3083.50	2392	3094.14	2438	3108.34	2486	3124.42	2546	3138.72	2621	3158.38
2349	3083.74	2393	3094.24	2439	3108.52	2487	3124.66	2547	3138.89	2622	3159.26
2350	3083.87	2394	3094.38	2440	3108.72	2488	3124.88	2549	3139.26	2623	3164.04
2351	3083.96	2395	3094.46	2441	3109.10	2489	3124.94	2550	3139.36	2624	3164.20
2353	3084.15	2396	3094.53	2442	3109.14	2490	3125.12	2557	3140.98	2625	3164.41
2354	3084.35	2397	3094.64	2443	3109.33	2493	3125.56	2558	3141.71	2628	3164.96
2355	3084.48	2399	3095.79	2444	3109.67	2494	3125.68	2559	3142.66	2629	3165.48
2355a	3084.81	2400	3095.95	2445	3109.79	2495	3125.72	2560	3142.77	2630	3165.61
2355b	3084.87	2401	3096.29	2446	3109.90	2496	3126.01	2561	3142.88	2635	3167.90
2355c	3084.92	2402	3096.66	2447	3110.33	2497	3126.44	2562	3143.98	2636	3168.25
2355d	3084.99	2403	3096.92	2448	3110.48	2498	3126.81	2563	3144.23	2637	3173.34
2356	3085.11	2404	3097.04	2449	3110.65	2499	3126.89	2564	3144.97	2638	3174.55
2357	3085.15	2405	3098.30	2451	3111.32	2501	3127.75	2567	3145.62	2642	3181.97
2358	3085.57	2406	3098.36	2452	3111.74	2502	3127.91	2568	3145.70	2643	3182.20
2360	3087.19	2407	3098.52	2453	3111.91	2503	3127.97	2569	3145.95	2650	3184.83
2361	3087.27	2408	3098.66	2454	3112.03	2504	3128.53	2570	3146.41	2651	3185.24
2362	3087.34	2409	3098.80	2455	3112.13	2505	3128.59	2571	3146.78	2652	3191.29
2363	3087.44	2410	3098.88	2456	3112.20	2506	3128.92	2572	3146.89	2653	3191.36
2364	3087.55	2410a	3099.14	2457	3112.39	2507	3129.05	2575	3147.25	2654	3191.70
2365	3087.66	2411	3099.28	2458	3112.77	2508	3129.18	2576	3147.40	2655	3192.00
2365a	3087.71	2412	3099.34	2459	3114.50	2513	3130.34	2577	3147.53	2656	3196.09
2366	3087.82	2413	3099.50	2461	3114.50	2519	3132.09	2581	3148.14	2657	3197.84
2367	3088.14	2414	3099.69	2462	3114.71	2520	3132.25	2594	3149.77	2658	3199.57
2368	3088.40	2415	3099.81	2463	3114.86	2523	3133.06	2595	3150.54	2659	3199.67
2369	3088.68	2416	3099.98	2464	3114.93	2524	3134.52	2596	3150.63	2660	3199.69
2369a	3088.77	2417	3100.05	2465	3115.27	2525	3134.64	2597	3150.76	2661	3200.26
2370	3088.86	2418	3100.35	2466	3115.42	2526	3134.95	2598	3151.34		
2371	3088.90	2419	3100.65	2467	3115.88	2527	3135.04	2599	3153.05		
2372	3089.04	2420	3100.72	2468	3117.42	2528	3135.09	2600	3153.19		
2373	3089.75	2421	3101.17	2469	3117.90	2529	3135.28	2601	3153.44		
2374	3089.90	2422	3102.51	2470	3118.12	2530	3135.42	2602	3153.56		
2375	3090.01	2423	3103.06	2471	3118.27	2531	3135.58	2603	3154.78		
2376	3090.19	2424	3103.30	2472	3118.55	2532	3135.78	2604	3154.88		

**Table 2:** Vibration-rotation wavenumbers for the v_3_ fundamental band of methane at 3018.9 cm^−1^ at low pressures

*P*	cm^−1^	Line No.	*R*	cm^−1^	Line No.	*Q*	cm^−1^	Line No.
								
			0	3028.744	2110		3018.815	2020
							3018.649	2019n
1	3009.000	1975	1	3038.498	2160		3018.602	2019m
							3018.533	2019l
							3018.349	2019k
2	2990.057	1886	2	3048.180	2188		3018.230	2019j
	2999.000	1885					3018.210	2019i
							3017.874	2019h
3	2989.045	1845d	3	3057.692	2231b_1_		3017.820	2019g
	2988.945	1845c		3057.739	2231b_2_		3017.748	2019f
	2978.800	1845b					3017.708	2019e
							3017.453	2019d
4	2979.033	1800d	4	3067.167	2256a		3017.325	2019c
	2978.937	1800c		3067.266	2256b		3017.245	2019b
	2978.872	1800b					3017.167	2019a
	2988.656	1800a					3016.930	2019
							3016.730	2018f
5	2968.861	1743d	5	3076.576	2310		3016.640	2018e
	2968.748	1743c		3076.696	2310a		3016.573	2018d
	2968.456	1743b		3076.738	2311		3016.480	2018c
	2968.404	1743a					3016.384	2018b
							3016.304	2018a
6	2958.636	1680p	6	3085.862	2358a		3015.978	2018
	2958.508	1680o		3086.046	2358b_1_		3015.790	2017b
	2958.430	1680n		3086.070	2358b_2_		3015.660	2017a
	2958.210	1680m					3015.549	2017
	2958.109	16801					3015.208	2016f
	2958.009	1680k						
7	2948.465	1632	7	3095.110	2397a			
	2948.405	1631		3095.174	2397b			
	2948.230	1629		3095.368	2397c			
	2948.120	1628		3095.680	2398			
	2948.081	1627						
	2947.888	1625						
	2947.792	1624						
	2947.660	1623						
	2947.415	1622						
8	2938.222	1569	8	3104.248	2427a			
	2938.168	1568		3104.309	2427b			
	2937.928	1566		3104.374	2427c			
	2937.900	1565		3104.598	2427d			
	2937.736	1564						
	2937.620	1563						
	2937.456	1562						
	2937.260	1560						
	2937.208	1559						
9	2927.932	1510	9	3113.300	2458a			
	2927.903	1509		3113.403	2458b			
	2927.378	1506		3113.423	2458b_1_			
	2927.342	1505		3113.720	2458c_2_			
	2927.025	1503						
	2926.837	1501						
	2926.750	1500						
	2926.670	1499						
10	2917.610	1451	10	3122.272	2482a			
	2917.032	1447		3122.382	2482b			
	2916.938	1446		3122.435	2482c			
	2916.730	1445		3122.775	2483			
	2916.480	1444						
	2916.373	1443						
	2916.293	1442						
	2916.173	1441						
	2915.983	1440						
11	2907.285	1390	11	3131.197	2515			
	2906.688	1385		3131.259	2515a			
	2906.592	1384		3131.382	2516			
	2906.560	1383		3131.749	2518			
	2906.232	1381						
	2906.062	1380						
	2905.770	1377						
	2905.647	1376						
	2905.507	1375						
12	2896.932	1333	12	3140.028	2551			
	2896.253	1330		3140.107	2552			
	2896.157	1329		3140.235	2553			
	2895.765	1325		3140.320	2554			
	2895.705	1324		3140.515	2555			
	2895.187	1320		3140.660	2556			
	2895.078	1319						
	2895.000	1318						
	2894.960	1317						
13	2886.570	1270	13	3148.815	2587			
	2886.070	1268		3149.010	2588a			
	2885.813	1266		3149.050	2588b			
	2885.718	1265		3149.095	2589			
	2885.310	1262		3149.345	2591			
	2885.190	1261		3149.423	2592			
	2885.050	1260		3149.480	2593			
	2884.867	1258						
	2884.720	1257						
	2884.620	1256						
	2884.463	1255						
	2884.413	1254						
	2884.348	1253						
14	2876.182	1205	14	3157.470	2616			
	2875.622	1202		3157.660	2617			
	2875.402	1200		3157.730	2618			
	2875.368	1199		3158.070	2619			
	2874.937	1195						
	2874.873	1194						
	2874.783	1193						
	2874.693	1192						
	2874.558	1190						
	2874.477	1189						
	2873.790	1185						
	2873.723	1184						
	2873.695	1183						
	2873.603	1182						
15	2865.770	1147	15	3166.040	2631			
	2865.340	1146		3166.270	2632			
	2864.952	1144		3166.490	2633			
	2864.683	1143		3166.720	2634			
	2864.403	1142						
	2864.213	1141						
	2863.960	1140						
	2863.890	1139						
	2863.110	1138						
	2863.040	1137						
	2863.005	1136						
	2862.930	1135						
16	2854.577	1112	16	3174.80	2639			
	2854.507	1111		3175.22	2640			
	2854.137	1110		3178.08	2641			
	2853.912	1109						
	2853.863	1108						
	2853.663	1107						
	2853.420	1106						
	2853.310	1105						
	2853.217	1104						
	2852.377	1102						
	2852.310	1101						
			17	3183.01	2644			
				3183.14	2645			
				3183.23	2646			
				3183.37	2647			
				3183.46	2648			
				3183.66	2649			

## Footnotes

2 Figures in brackets indicate the literature references at the end of this paper.
